# Supplementary material for: A Simple and Easily Implemented Method for the Regioselective Introduction of Deuterium into Azolo[1,5-a]pyrimidines Molecules
Source: Molecules. 2023 Mar 22;28(6):2869. doi: 10.3390/molecules28062869 (PMC10054722; doi:10.3390/molecules28062869)
Supplement: Supplementary file 1 [file molecules-28-02869-s001.zip › molecules-2240788-supplementary.pdf]

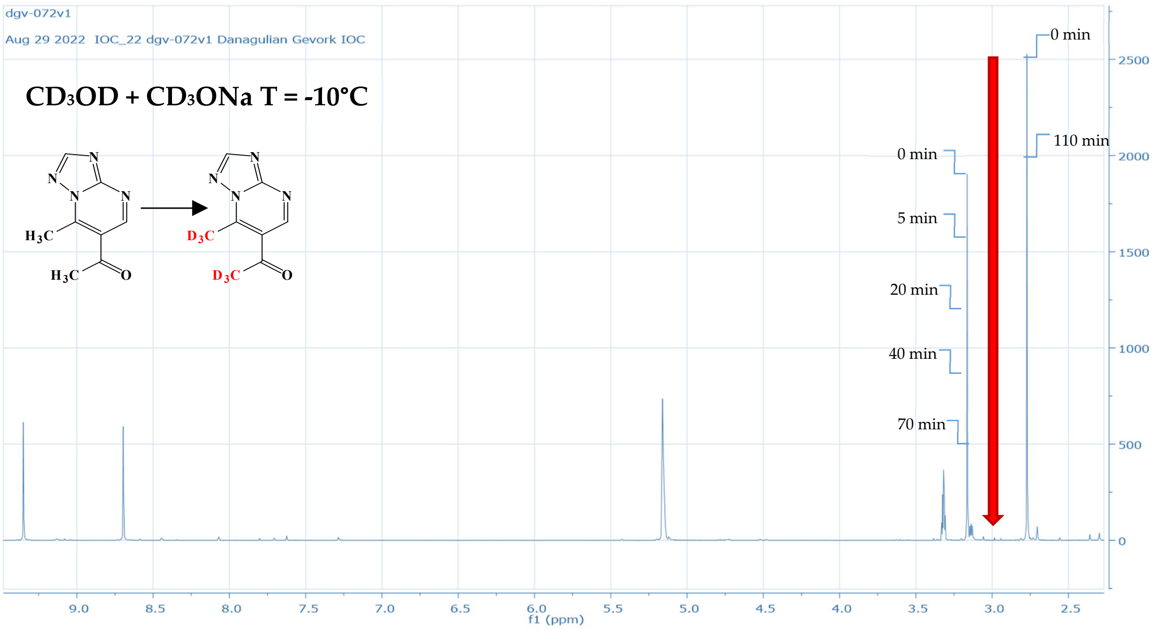

Scheme S1. Schematic representation of the dynamics of the decrease in the intensity of the peaks of methyl groups after the addition of CD_3OD/CD_3ONa to 6-acetyl-7-methyl-1,2,4-triazolo[1,5-*a*]pyrimidine (**5**) in a time interval of 0-110 minutes at a temperature of (-10°C).

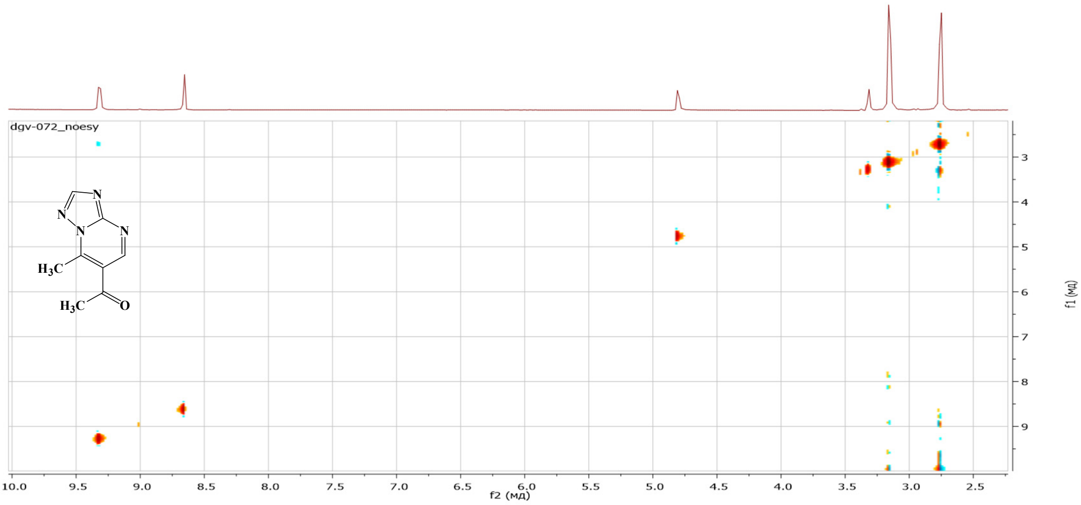

Figure S1. NOESY spectrum of 6-acetyl-7-methyl-1,2,4-triazolo [1,5-*a*]pyrimidine **5.**

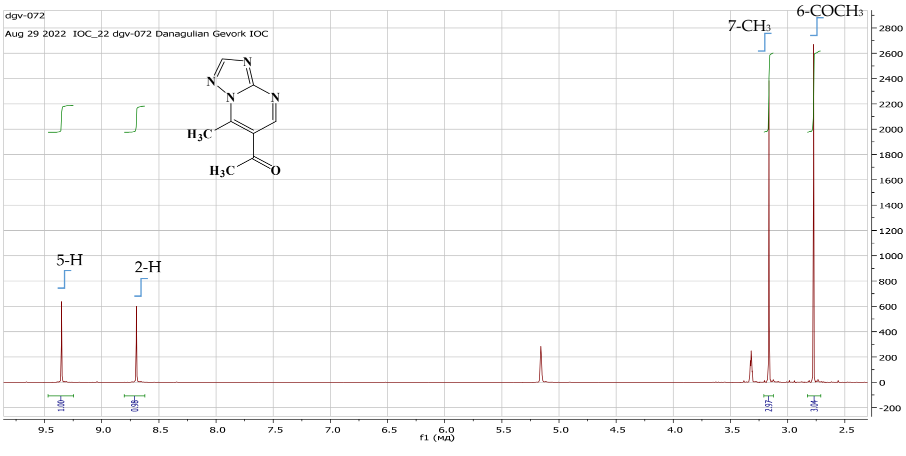

Figure S2. 6-Acetyl-7-methyl-1,2,4-triazolo[1,5-*a*]pyrimidine **5,** T = 30°C.

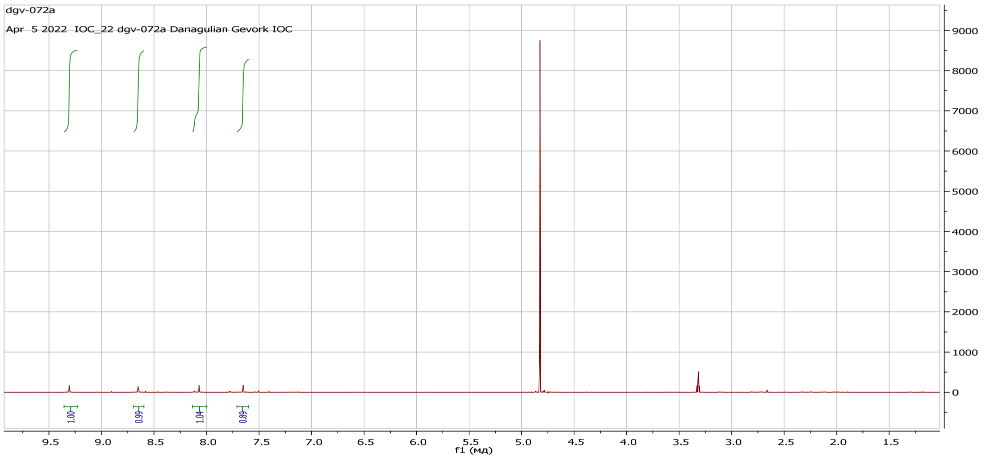

Figure S3. 6-d_3-Acetyl-7-d_3-methyl-1,2,4-triazolo[1,5-*a*]pyrimidine **6,** T = 30°C.

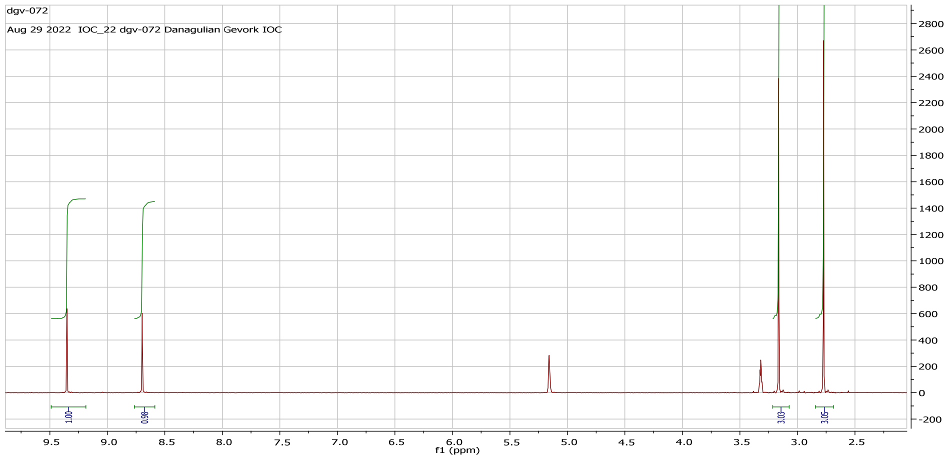

Figure S4. 6-Acetyl-7-methyl-1,2,4-triazolo[1,5-*a*]pyrimidine **5,** T = -10°C.

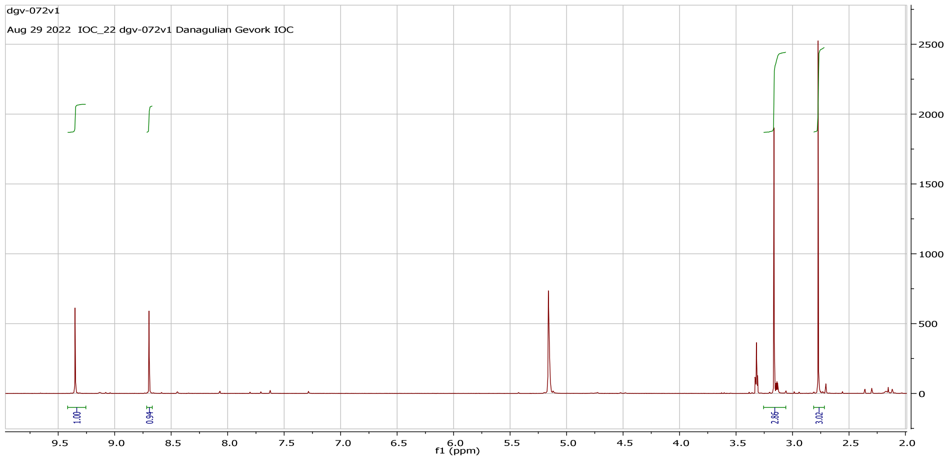

Figure S5. 6-d_3-Acetyl-7-d_3-methyl-1,2,4-triazolo[1,5-*a*]pyrimidine **6**, *T* = -10°C after 5 min.

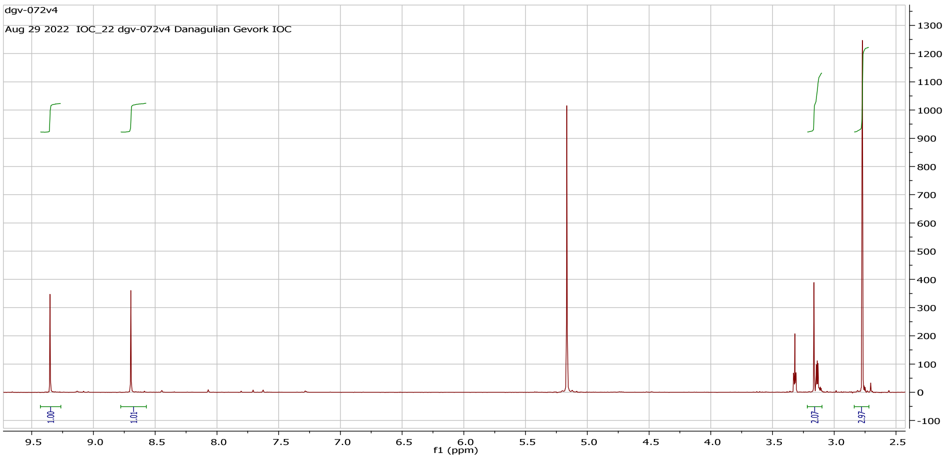

Figure S6. 6-d_3-Acetyl-7-d_3-methyl-1,2,4-triazolo[1,5-*a*]pyrimidine **6**, *T* = -10°C after 20 min.

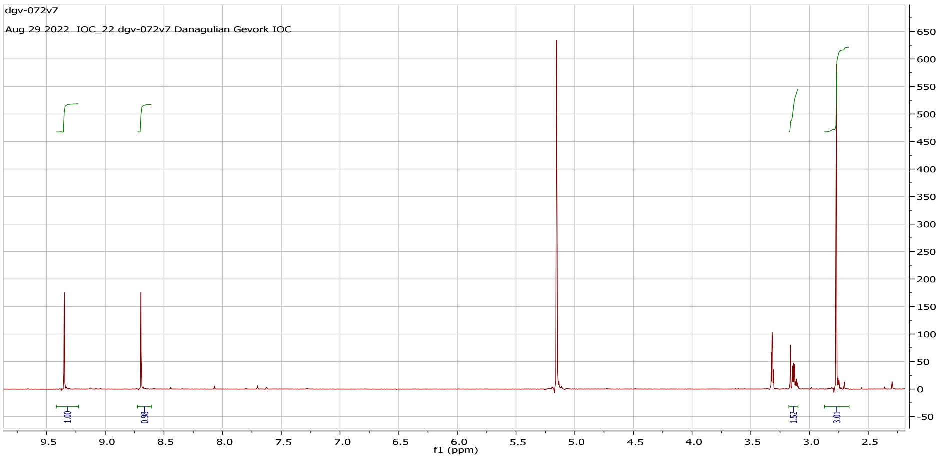

Figure S7. 6-d_3-Acetyl-7-d_3-methyl-1,2,4-triazolo[1,5-*a*]pyrimidine **6**, *T* = -10°C after 40 min.

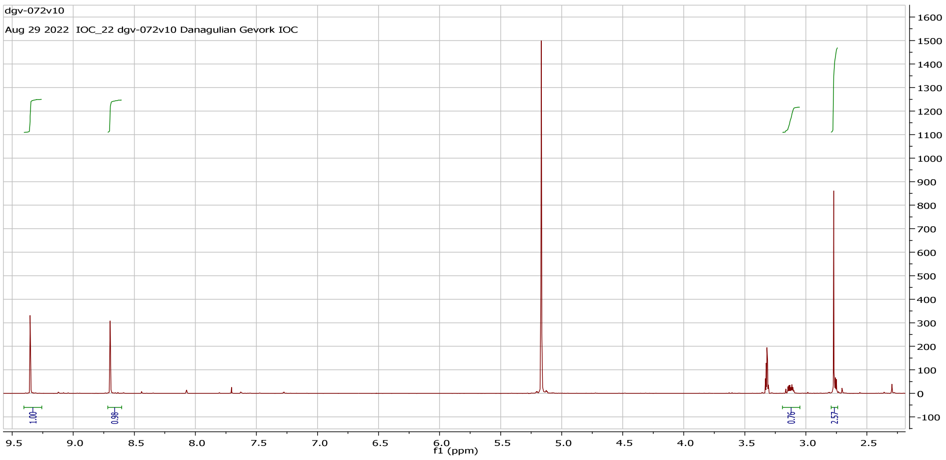

Figure S8. 6-d_3-Acetyl-7-d_3-methyl-1,2,4-triazolo[1,5-*a*]pyrimidine **6**, *T* = -10°C after 70 min.

Table S1. Kinetic study of the deuterium exchange from compound **5** to 6-d_3-acetyl-7-d_3-methyl-1,2,4-triazolo[1,5-*a*]pyrimidine **6** in CD_3OD + CD_3ONa at T = -10°C.

Time (min)	^{1}H-NMR (300 MHz, δ, ppm):
5	2.78 (s, 3.02H); 3.18 (s, 2.86H); 8.62 (s, 1H); 9.25 (s, 1H)
20	2.78 (s, 2.97H); 3.18 (s, 2.07H); 8.62 (s, 1H); 9.25 (s, 1H)
40	2.78 (s, 3.01H); 3.18 (s, 1.52H); 8.62 (s, 1H); 9.25 (s, 1H)
70	2.78 (s, 2.57H); 3.18 (s, 0.76H); 8.62 (s, 1H); 9.25 (s, 1H)

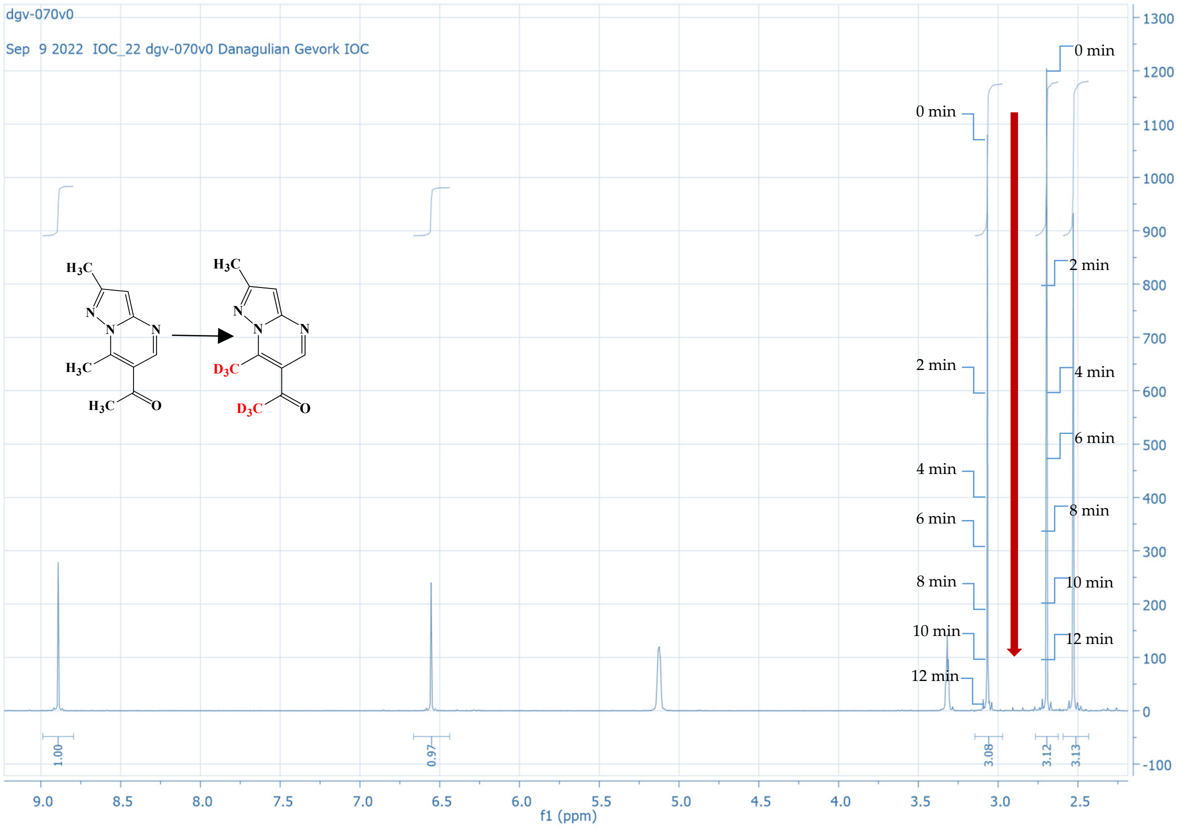

Scheme S2. Schematic representation of the dynamics of the decrease in the intensity of the peaks of methyl groups after the addition of CD_3OD/CD_3ONa to 2,7-dimethyl-6-acetylpyrazolo[1,5-*a*]pyrimidine (**1**) in a time interval of 0-12 minutes at a temperature of (-15°C).

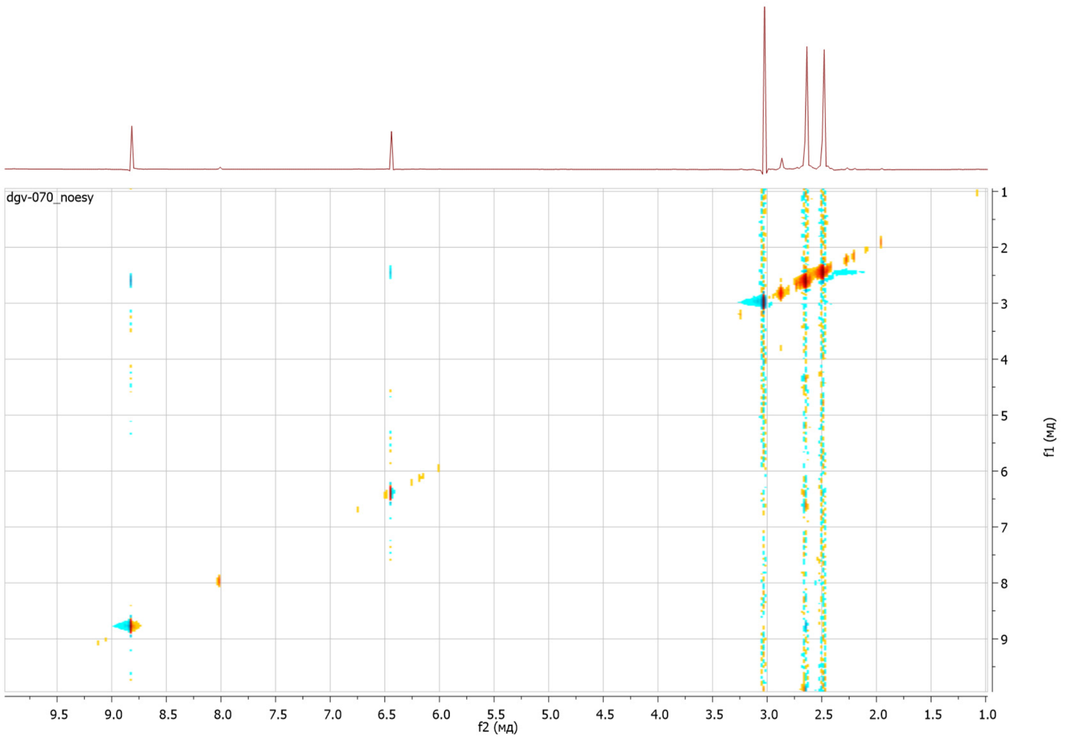

Figure S9. NOESY spectrum of 2,7-dimethyl-6-acetylpyrazolo[1,5-*a*]pyrimidine **1**.

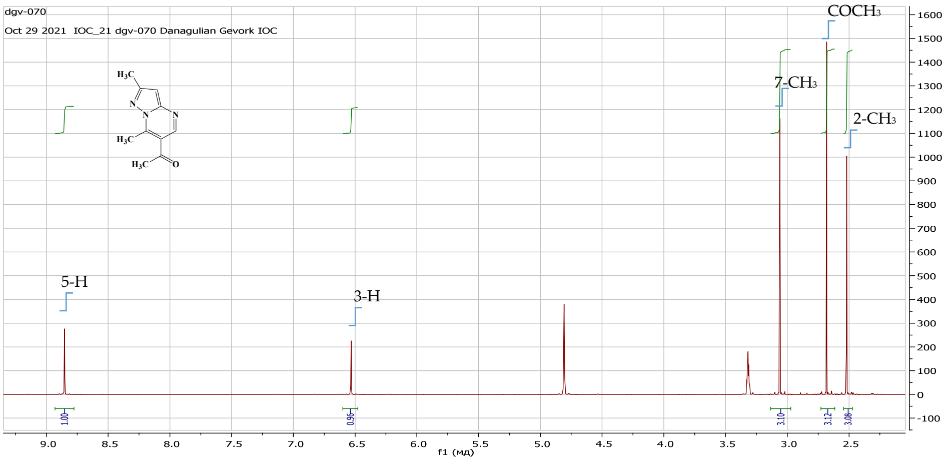

Figure S10. 6-Acetyl-2,7-dimethylpyrazolo[1,5-*a*]pyrimidine **1**, T = 30°C.

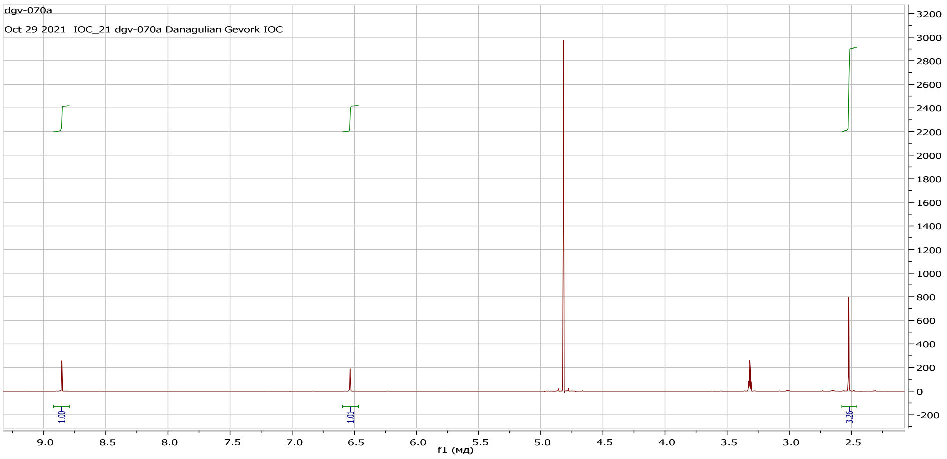

Figure S11. 6-d3-Acetyl-7-d3-methyl-2-methylpyrazolo[1,5-*a*]pyrimidine **3**, T = 30°C.

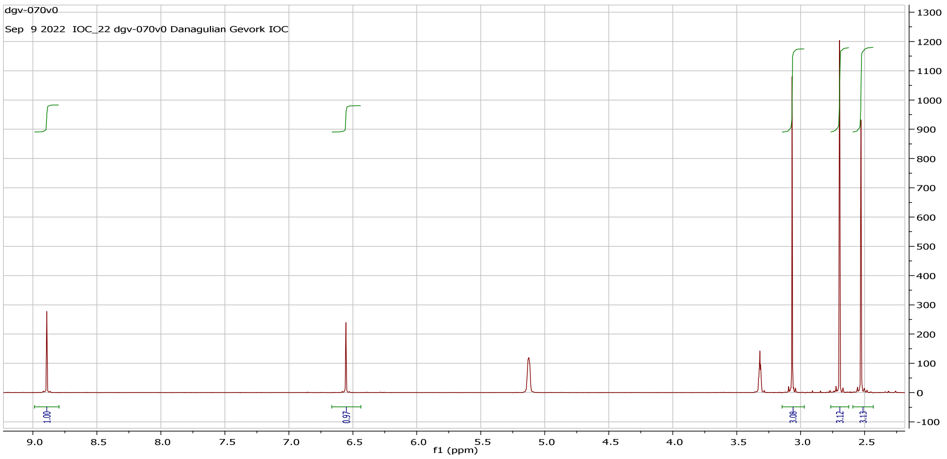

Figure S12. 6-Acetyl-2,7-dimethylpyrazolo[1,5-*a*]pyrimidine **1**, *T* = -15°C.

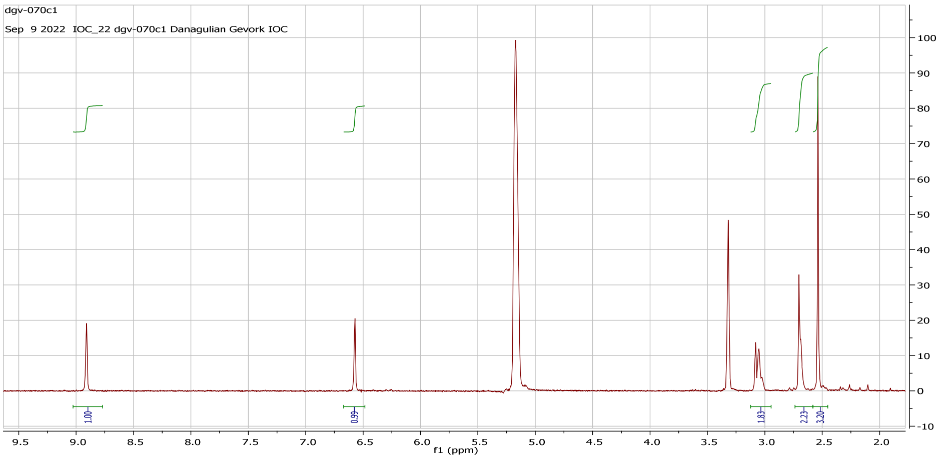

Figure S13. 6-d3-Acetyl-7-d3-methyl-2-methylpyrazolo[1,5-*a*]pyrimidine **3**, *T* = -15°C after 2 min.

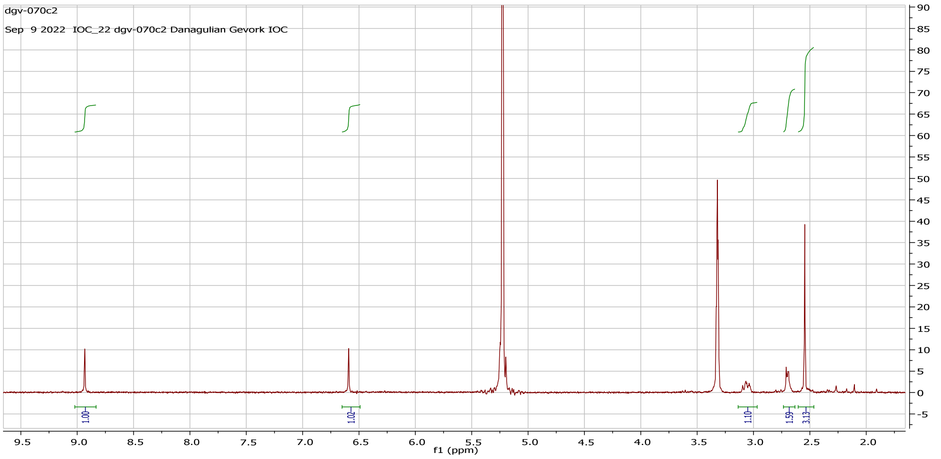

Figure S14. 6-d3-Acetyl-7-d3-methyl-2-methylpyrazolo[1,5-*a*]pyrimidine **3**, *T* = -15°C after 4 min.

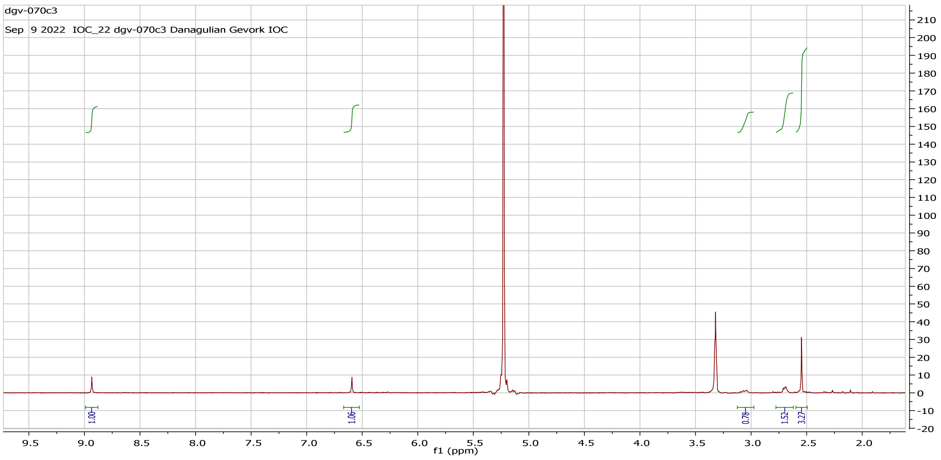

Figure S15. 6-d3-Acetyl-7-d3-methyl-2-methylpyrazolo[1,5-*a*]pyrimidine **3**, *T* = -15°C after 6 min.

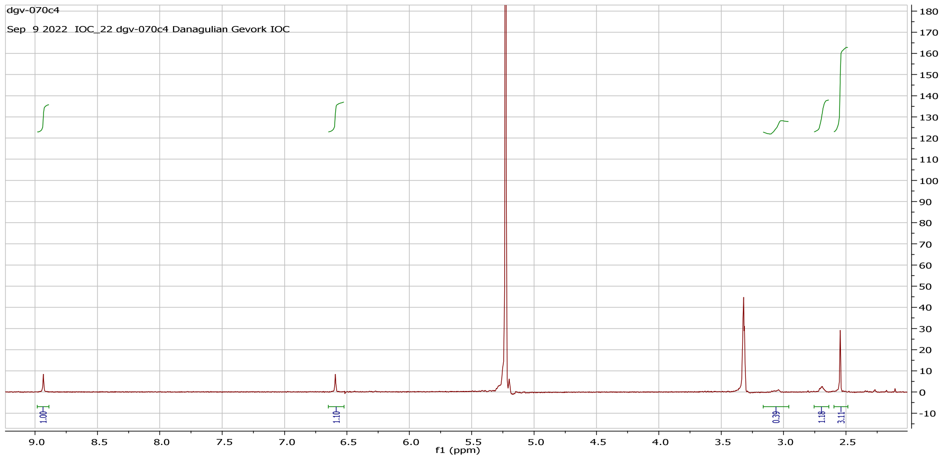

Figure S16. 6-d_3-Acetyl-7-d_3-methyl-2-methylpyrazolo[1,5-*a*]pyrimidine **3**, *T* = -15°C after 8 min.

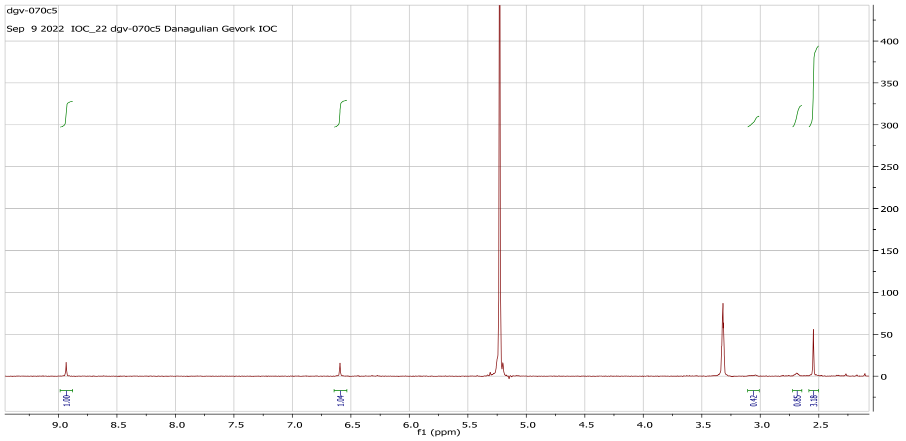

Figure S17. 6-d_3-Acetyl-7-d_3-methyl-2-methylpyrazolo[1,5-*a*]pyrimidine **3**, *T* = -15°C after 10 min.

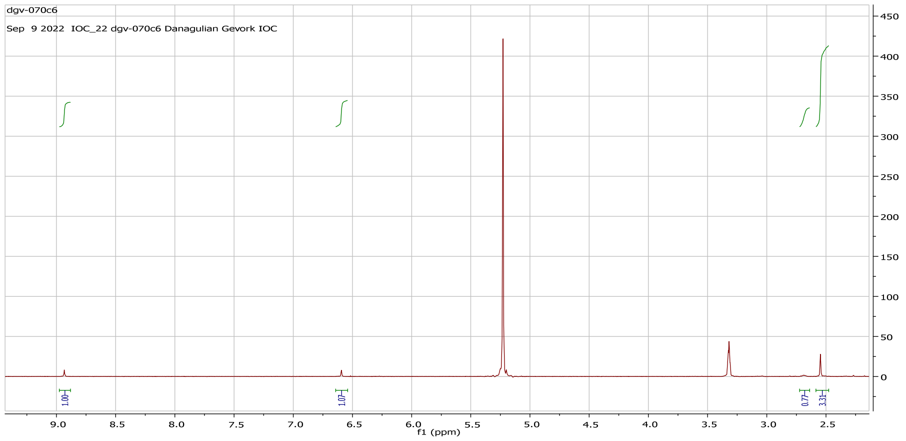

Figure S18. 6-d_3-Acetyl-7-d_3-methyl-2-methylpyrazolo[1,5-*a*]pyrimidine **3**, *T* = -15°C after 12 min.

Table S2. Kinetic study of the deuterium exchange from compound **1** to 6-d_3-acetyl-7-d_3-methyl-2-methylpyrazolo[1,5-*a*]pyrimidine **3** in CD_3OD + CD_3ONa at T = -15°C.

Time (min)	^{1}H-NMR (300 MHz, δ, ppm):
2	2.50 (s, 3H); 2.65 (s, 2.23H); 3.14 (s, 1.83H); 6.55 (s, 1H); 8.82 (s, 1H)
4	2.50 (s, 3H); 2.65 (s, 1.59H); 3.14 (s, 1.1H); 6.55 (s, 1H); 8.82 (s, 1H)
6	2.50 (s, 3H); 2.65 (s, 1.52H); 3.14 (s, 0.78H); 6.55 (s, 1H); 8.82 (s, 1H)
8	2.50 (s, 3H); 2.65 (s, 1.18H); 3.14 (s, 0.39H); 6.55 (s, 1H); 8.82 (s, 1H)
10	2.50 (s, 3H); 2.65 (s, 0.45H); 3.14 (s, 0.0.42H); 6.55 (s, 1H); 8.82 (s, 1H)
12	2.50 (s, 3H); 2.65 (s, 0.77H); 3.14 (s, 0.0H); 6.55 (s, 1H); 8.82 (s, 1H)

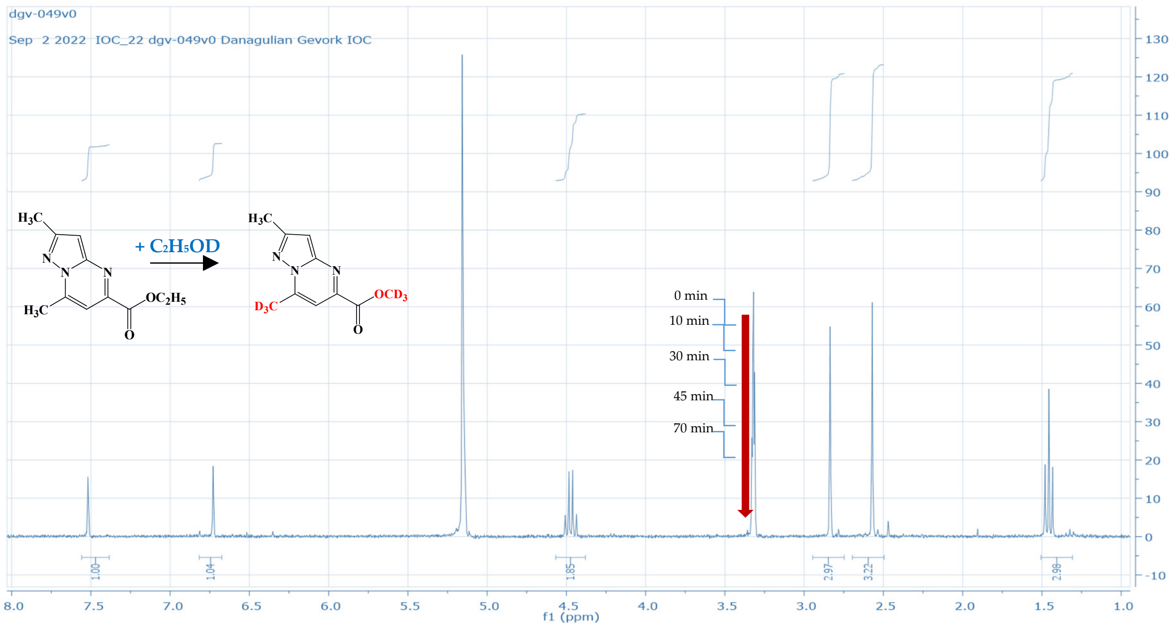

Scheme S3. Schematic representation of the dynamics of the decrease in the intensity of the peaks of methyl groups after the addition of CD_3OD/CD_3ONa to 2,7-dimethyl-5-ethoxycarbonylpyrazolo[1,5-*a*]pyrimidine (**10**) in a time interval of 0-70 minutes at a temperature of (-10°C).

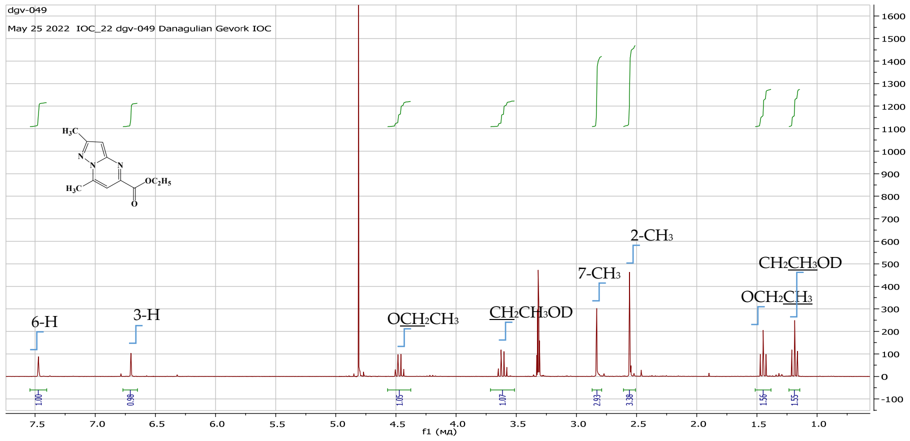

Figure S20. 2,7-Dimethyl-5-ethoxycarbonylpyrazolo[1,5-*a*]pyrimidine **10**, T = 30°C.

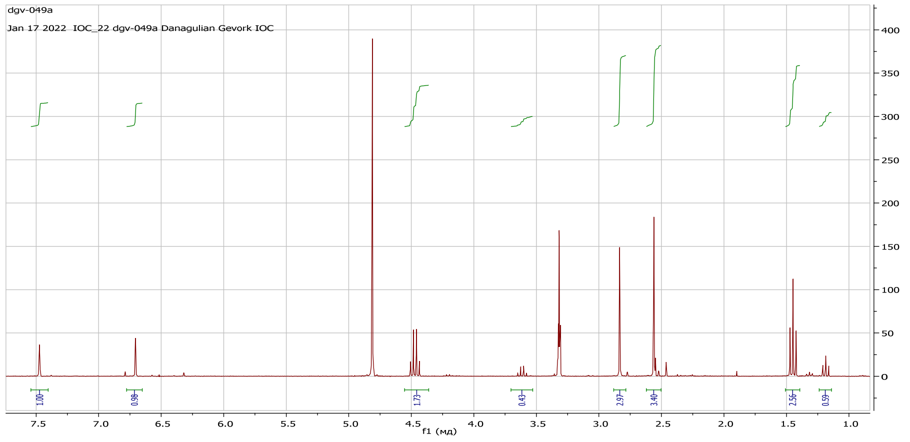

Figure S21. 2,7-Dimethyl-5-ethoxycarbonylpyrazolo[1,5-*a*]pyrimidine **10**, T = 30°C.

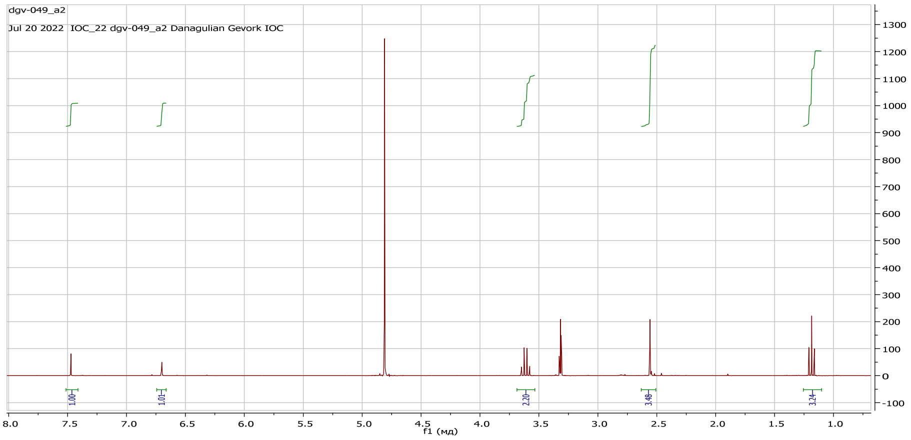

Figure S22. 7-d$_3$-Methyl-2-methyl-5-d$_3$-metoxycarbonylpyrazolo[1,5-*a*]pyrimidine **10**, T = 30°C.

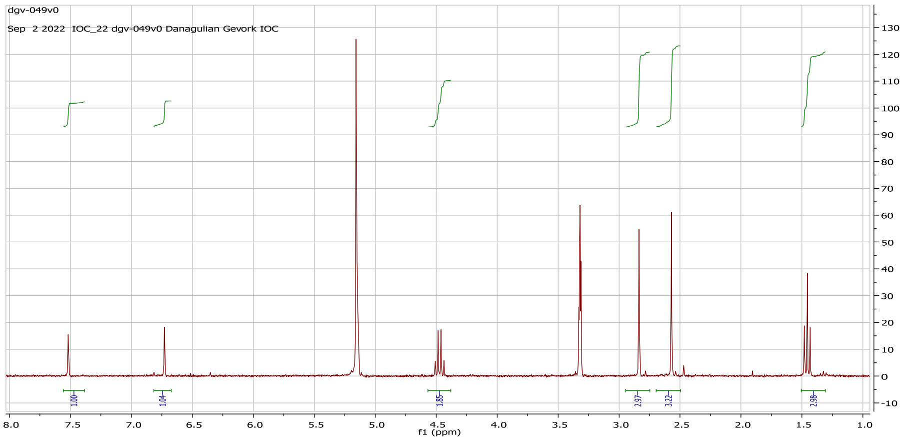

Figure S23. 2,7-Dimethyl-5-ethoxycarbonylpyrazolo[1,5-*a*]pyrimidine **10**, T= -10°C.

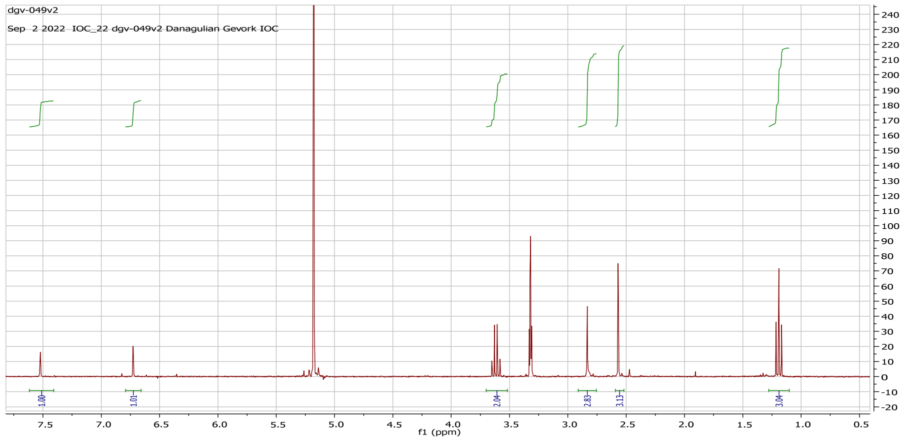

Figure S24. 7-d$_3$-Methyl-2-methyl-5-d$_3$-metoxycarbonylpyrazolo[1,5-*a*]pyrimidine **14**, T = -10°C after 10 min.

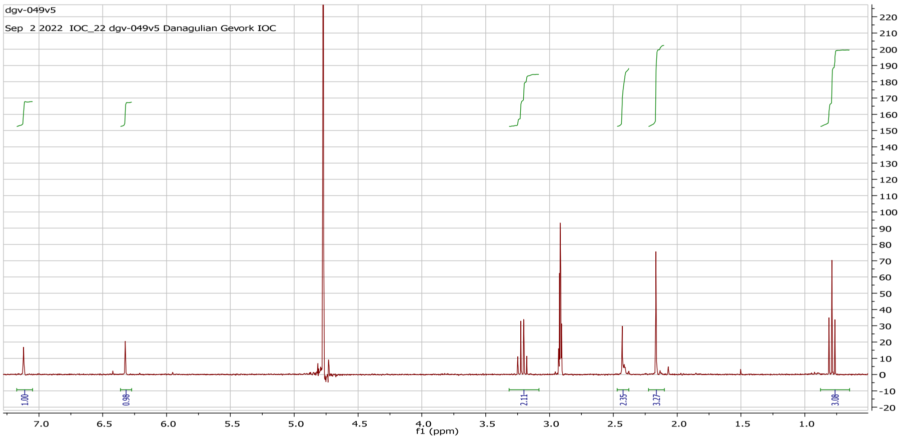

Figure S25. 7-d3-Methyl-2-methyl-5-d3-metoxycarbonylpyrazolo[1,5-*a*]pyrimidine **14**, T = -10°C after 30 min.

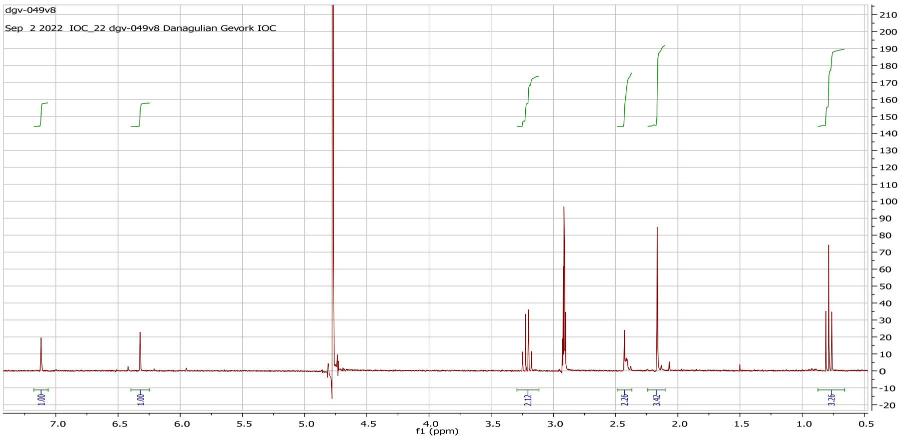

Figure S26. 7-d3-Methyl-2-methyl-5-d3-metoxycarbonylpyrazolo[1,5-*a*]pyrimidine **14**, T = -10°C after 45 min.

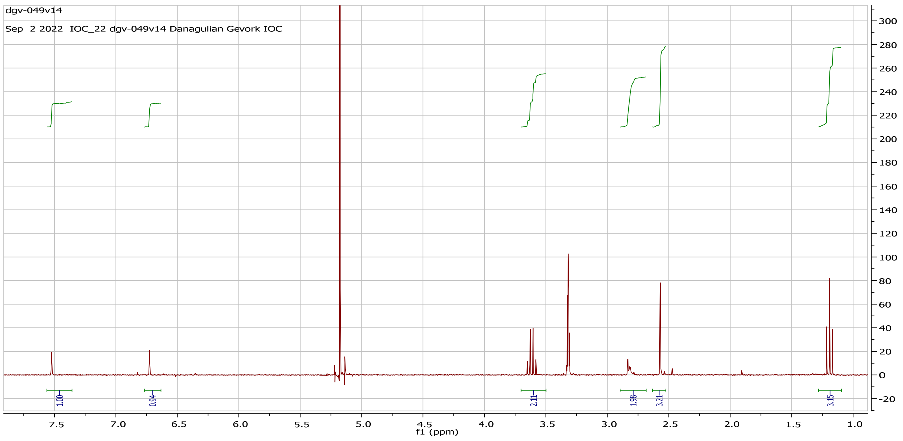

Figure S27. 7-d3-Methyl-2-methyl-5-d3-metoxycarbonylpyrazolo[1,5-*a*]pyrimidine **14**, T = -10°C after 70 min.

Table S3. Kinetic study of the deuterium exchange from compound **10** to 7-d_3-methyl-2-methyl-5-d_3-metoxycarbonylpyra-zolo[1,5-*a*]pyrimidine **14** in CD_3OD + CD_3ONa at T = -10°C.

Time (min)	^{1}H-NMR (300 MHz, δ, ppm):
5	1.2 (t, 3H); 2.55 (s, 3H); 3.6 (q, 2H); 6.7 (s, 1H); 7.45 (s, 1H)
10	1.42(t, 3H); 2.55 (s, 3H); 2.82 (s, 2.83H); 4.45 (q, 2H); 6.7 (s, 1H); 7.45 (s, 1H)
30	1.42(t, 3H); 2.55 (s, 3H); 2.82 (s, 2.35H); 4.45 (q, 2H); 6.7 (s, 1H); 7.45 (s, 1H)
45	1.42(t, 3H); 2.55 (s, 3H); 2.82 (s, 2.26H); 4.45 (q, 2H); 6.7 (s, 1H); 7.45 (s, 1H)
70	1.42(t, 3H); 2.55 (s, 3H); 2.82 (s, 1.98H); 4.45 (q, 2H); 6.7 (s, 1H); 7.45 (s, 1H)
